# Dynamics in a behavioral–epidemiological model for individual adherence to a nonpharmaceutical intervention

**DOI:** 10.1073/pnas.2311584120

**Published:** 2023-10-27

**Authors:** Chadi M. Saad-Roy, Arne Traulsen

**Affiliations:** ^a^Miller Institute for Basic Research in Science, University of California, Berkeley, CA 94720; ^b^Department of Integrative Biology, University of California, Berkeley, CA 94720; ^c^Department of Theoretical Biology, Max Planck Institute for Evolutionary Biology, Plön 24306, Germany

**Keywords:** individual decision-making, behavioral–epidemiological dynamics, nonpharmaceutical interventions

## Abstract

The SARS-CoV-2 pandemic has highlighted the importance of behavioral drivers in epidemic dynamics. With the relaxation of mandated nonpharmaceutical interventions (NPIs) formerly in place to decrease transmission, such as mask-wearing or social distancing, adherence to an NPI is now the result of individual decision-making. To study these coupled dynamics, we embed a game-theoretic model for individual NPI adherence within an epidemiological model. When the disease is endemic, we find that our model has multiple (but none concurrently stable) equilibria: one each with zero, complete, or partial NPI adherence. Surprisingly, for the equilibrium with partial NPI adherence, the number of infections is independent of the transmission rate. Therefore, in that regime, a change in the rate of pathogen transmission, e.g., due to another (mandated) NPI or a new variant, has no effect on endemic infection levels. On the other hand, we show that vaccination successfully decreases endemic infection levels, and, unexpectedly, also reduces the number of susceptibles at equilibrium when there is partial adherence. From a game-theoretic perspective, we find that highly effective NPIs lead at most to partial adherence. As this effectiveness decreases, partially effective NPIs initially lead to increases in population-level adherence, especially if the risk is high enough. However, a completely ineffective NPI results in no adherence. Furthermore, we identify parameter regions where the individual incentives may not align with those of society as a whole. Overall, our findings illustrate complexities that can arise due to behavioral–epidemiological feedback and suggest appropriate measures to avoid more pessimistic population-level outcomes.

## Significance

While SARS-CoV-2 transmission continues, adherence to nonpharmaceutical interventions (NPIs) is now largely based on individual decision-making, which itself depends on infection levels, adherence cost, and perceived risk of an infection. With a cross-scale behavioral–epidemiological model, we find that when partial adherence is the stable outcome, the transmission rate has no influence on endemic infection levels because individuals tend to adhere less to NPIs when infection levels decrease (and vice versa). However, we show that vaccination decreases infecteds and susceptibles in this setting. Furthermore, we find that a very effective NPI is only partially adhered to, whereas moderate efficacy can lead to complete adherence. Overall, our results illustrate the importance of vaccination and of the rational deployment of additional NPIs.

The SARS-CoV-2 pandemic has caused significant morbidity and mortality, with over 676 million confirmed cases and 6.8 million fatalities as of March 10 2023 (1). At the beginning of the pandemic, nonpharmaceutical interventions (NPIs), such as mask-wearing, school closures, and shelter-in-place orders were mandated in many regions to decrease transmission, e.g., refs. 2 and 3. Other regions relied instead on individual decision-making to choose for themselves whether to adhere to various NPIs. Since these early days, multiple effective vaccines against SARS-CoV-2 have been developed (4–6). Their subsequent successful deployment across the world then led many jurisdictions to remove mandated NPIs, thus leaving individuals to personally decide on NPI adherence based on their preference. Throughout the pandemic, a number of salient epidemiological unknowns were investigated via a combination of modelling, data, and statistical inference (7). A remaining outstanding issue is the incorporation of behavioral dynamics for individual-based NPI adherence in epidemiological models.

A number of previous studies have examined the impact of NPIs on pathogen dynamics. For example, Baker et al. (8) used SIRS models to show that the long-term epidemiological effect of a decrease in transmission rate depends on the characteristics of the pathogen. In particular, for pathogens with high basic reproduction numbers, a decrease in transmission rate leads to transient effects and no impact on long-term dynamics. For medium-term dynamics, other work (9) examined a simple SIRS-like immuno-epidemiological model for potential SARS-CoV-2 future scenarios. These authors found that seasonality, combined with a period of decreased transmission and stronger immunity, can lead to a delayed, but bigger, “second” epidemic peak, whereas other NPI scenarios could give different outcomes.

Other studies have coupled social and epidemiological dynamics, such as incorporation of behavioral heterogeneity (10), social distancing (11), social norms (12), school-closures (13), adaptive behavior (14), awareness (15), or vaccination decision-making (16, 17) into epidemiological dynamics (for more examples, see, e.g., ref. 18 and references therein). Together, these studies illustrate the rich dynamical behavior that can emerge in light of various behavioral complications. In related work, the authors of ref. 19 studied the impacts of two contagions on the evolution of sociality. Finally, we have recently examined the rise of social dilemmas in a simple framework for adherence to an NPI, with either one or two choices for a particular NPI (e.g., kinds of face-mask) (20).

In general, individual decisions for adherence to an NPI are based on infection levels, the (potentially perceived) individual risk from infection, and the cost of adhering to the NPI. In prior work, we have assumed that there was a constant infection level to which individuals respond to (20). In reality, the decision-making of an individual is shaped by the cost of the NPI, the risk imposed by infection, and the probability of infection. This latter quantity is itself determined by epidemiological dynamics. In turn, the fraction of adherers then affects the transmission rate, giving rise to potential socio-epidemiological feedback. Here, we couple social and epidemiological dynamics to investigate the impacts of individual NPI adherence decision-making. With our model, we examine the potential long-term dynamics that arise from this coupling. Finally, we extend our model to examine the impact of vaccination on NPI decision-making.

## Model Framework

**Fig. 1.**
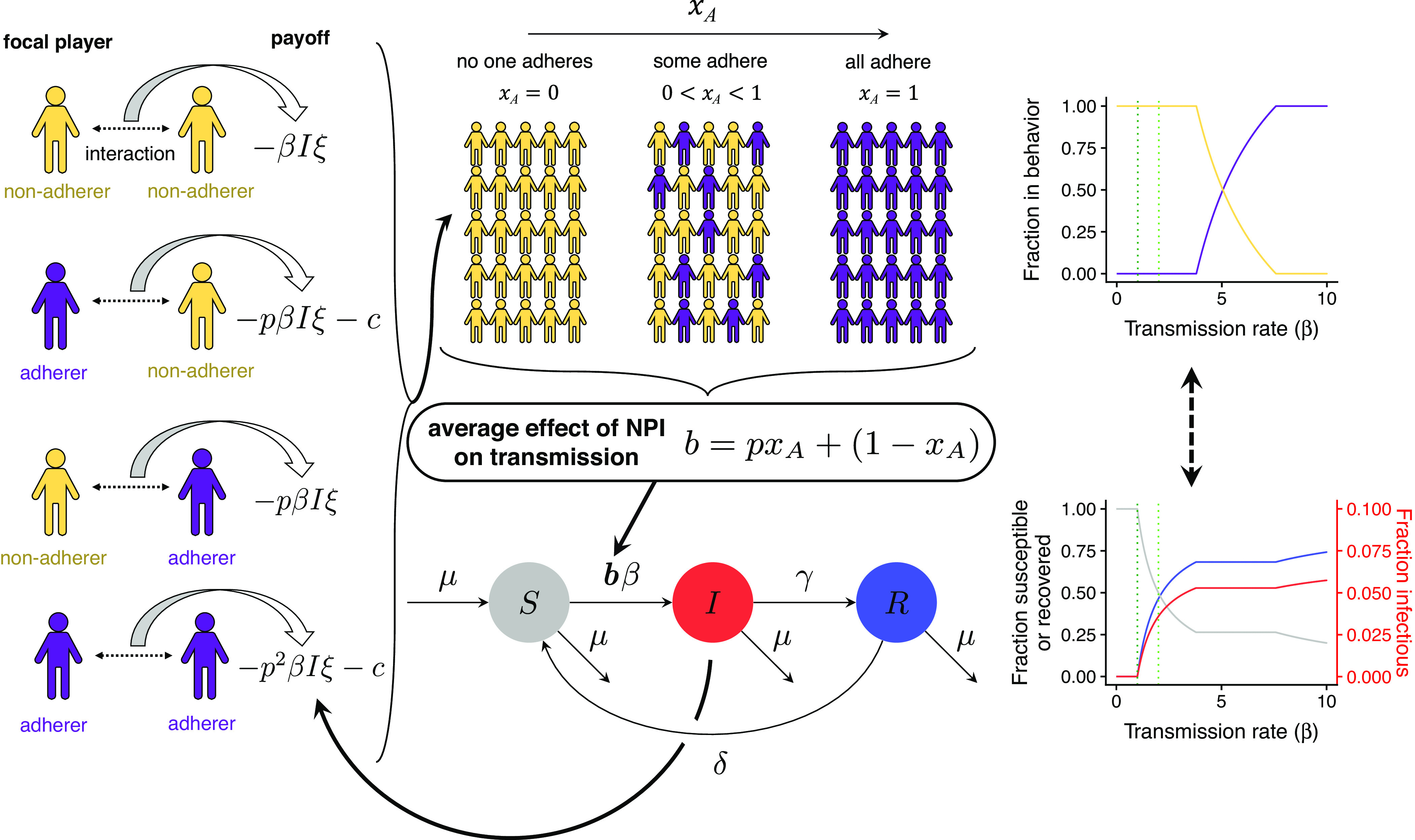
Model framework schematic. The *Left* panel depicts the payoff obtained from each kind of interaction. The *Middle* panels illustrate how the behavioral and epidemiological components are coupled. The *Right* panels are an example of the potential interplay between these dynamics as a function of the transmission rate β (per week), with parameter values: γ=1 per week, μ=0.02 per year, δ=4 per year, p=0.5, ξ=10, c=1 per week. The dotted lines indicate where $R_{0}=\frac{\beta }{\gamma +\mu }=1$ (dark green) and $pR_{0}=1$ (light green).

Previous studies with SIRS-like models have modelled the impact of NPIs as a decrease in the transmission rate β (see, e.g., refs. 8, 9, 21–25), so that the “new” transmission rate with the NPI is bβ, where 0≤b<1 is a measure of the effectiveness of the NPI. Here, we extend this approach and assume that NPI adherence is coupled with epidemiological dynamics. Our behavioral–epidemiological model is schematically illustrated in Fig. 1. We model b as a function of the fraction $x_{A}$ of individuals that are adhering to an NPI that decreases the transmission rate to pβ (i.e., p is the relative transmission rate and is thus a proxy for the ‘effectiveness’ of the NPI). Thus, this gives that

[1] $$\begin{array}{c} b=px_{A}+\left(1-x_{A}\right), \end{array}$$

where $x_{A}$ is the fraction of adherers A and $1-x_{A}$ is the fraction of nonadherers N. Thus, b is the average effect of the NPI on the transmission rate.

For the dynamics of $x_{A}$, we use the replicator equation as a model of social learning (17, 26–28). First, we define the payoffs for adherers and nonadherers. The individual payoffs in this two-player game depend on the transmission rate β, the fraction I of individuals that are infected, the relative probability of transmission p, the risk ξ one perceives the infection to pose, and the cost c of following the NPI. Furthermore, we assume that the NPI is symmetric, i.e., the relative transmission rate is p if either the focal player or the partner is adhering to the NPI. Thus, the respective payoffs are given by the matrix (20)[2]
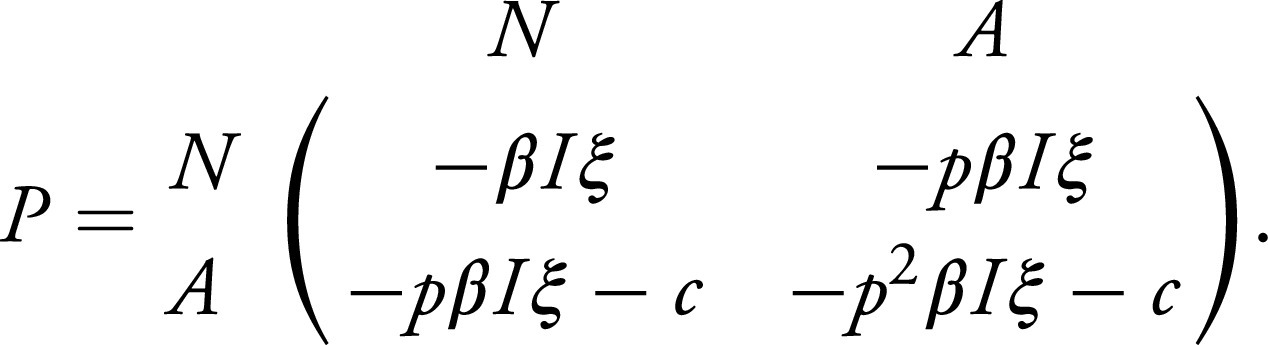


If a nonadherer is the focal player, the payoff when it interacts with another nonadherer is −βIξ, whereas if they interact with an adherer, the payoff is −pβIξ. Thus, their average payoff is $\pi_{N}=-p\beta I\xi x_{A}-\beta I\xi \left(1-x_{A}\right)$ On the other hand, if an adherer is the focal player, they pay a cost c to adhering. Therefore, if they interact with a nonadherer, the payoff is −pβIξ−c, whereas if they interact with an adherer, it is $-p^{2}\beta I\xi -c$. Thus, their payoff is $\pi_{A}=\left(-p^{2}\beta I\xi -c\right)x_{A}+\left(-p\beta I\xi -c\right)\left(1-x_{A}\right)$. Beyond the assumption that the NPI is symmetric, this formulation implicitly assumes that the perceived risk of the infection and the cost of adhering are constant across the population and over time. We assume that individuals switch to the strategy that yields a higher payoff in a fashion that depends linearly on payoff differences (17, 26). This leads to the differential equation $\frac{dx_{A}}{\mathrm{dt}}=x_{A}\left(1-x_{A}\right)\left(\pi_{A}-\pi_{N}\right)$.

To model epidemiological dynamics, we assume that the population size is constant and denote S as the fraction of individuals that are susceptible, I as the fraction of individuals that are infected, and R as the fraction of individuals that are recovered, so that S+I+R=1. Furthermore, we assume that μ is the birth (and death) rate, γ is the recovery rate, and δ is the rate of waning immunity. Finally, in our model, the NPI-affected transmission rate is $b\beta =[px_{A}+\left(1-x_{A}\right)]\beta$. Therefore, note that we implicitly assume that immunity and adherence are independent. Taking R=1−S−I, the coupled behavioral–epidemiological dynamics are then given by the following equations:

[3a] $$\begin{array}{cc} \frac{\mathrm{dS}}{\mathrm{dt}} & =\mu -\left[px_{A}+\left(1-x_{A}\right)\right]\beta SI-\mu S+\delta \left(1-S-I\right), \end{array}$$

[3b] $$\begin{array}{cc} \frac{\mathrm{dI}}{\mathrm{dt}} & =\left[px_{A}+\left(1-x_{A}\right)\right]\beta IS-\left(\gamma +\mu \right)I, \end{array}$$

[3c] $$\begin{array}{cc} \frac{dx_{A}}{\mathrm{dt}} & =x_{A}\left(1-x_{A}\right)\left(\pi_{A}-\pi_{N}\right), \end{array}$$

where $\pi_{N}=-\beta I\xi \left(1-x_{A}\right)-p\beta I\xi x_{A}$ and $\pi_{A}=p\pi_{N}-c$.

Note that implicit in our formulation is the fact that the individual choice of adherence to the NPI occurs continuously. Thus, $x_{A}$ could also be viewed as the probability of NPI adherence (see, e.g., refs. 28 and 29 for a discussion of a probabilistic perspective). Our model is therefore a proxy for the situation in which individuals choose a pure strategy.

## Results and Discussion

In the absence of disease, our model has two equilibria, with either zero or complete adherence (*Materials and Methods*). The stability of the former depends on whether the basic reproduction number (without control) is greater than 1 (*SI Appendix*). On the other hand, we find that complete adherence to the NPI is always unstable (*SI Appendix*). This intuitive result illustrates that even if an NPI could lead to local elimination if every individual adhered, such a state would not be stable if individuals are left to decide on their own adherence, i.e., without a “top-down” mandate. Note also that, in the absence of disease, no top-down regulation of an NPI is the most likely outcome.

As highlighted by the SARS-CoV-2 pandemic, local elimination is especially difficult for respiratory infections. Instead, these pathogens are often endemic. Furthermore, while the SARS-CoV-2 pandemic brought forth the potential short-term impact of disturbances on epidemiological dynamics due to various responses (refs. 22 and 30), an emerging focus has now been to understand the longer-term implications of continued intervention use (8). In this vein, we examine the potential long-term behavioral–epidemiological outcomes in our model when there are circulating infections.

We find that our model has up to three possible endemic equilibria (*Materials and Methods*): the classical SIRS endemic equilibrium with no one following the NPI, the classical SIRS endemic equilibrium, but with everyone following the NPI, and, most interestingly, an endemic equilibrium with partial adherence. In this latter setting (with partial adherence), we surprisingly find that the endemic level of infections does not depend on the transmission rate β (note that the fraction of individuals that are susceptible is also independent of β). Thus, a change in the transmission rate has no impact on the endemic infection levels at equilibrium, but it does affect the equilibrium level of adherence to the NPI. The intuition behind this result is as follows: If the transmission rate decreases, individuals perceive a decrease in the risk of infection, which leads to behavioral adjustments that compensate for the change in the transmission rate. This result has analogies to the classic effect that vaccination below the elimination threshold has no (or at most a negligible) effect on the susceptibles at the endemic equilibrium (see ref. 31 and references therein for a brief review and discussion).

Overall, this lack of dependence on the transmission rate yields important insight into the design of NPIs to decrease infection levels. If there is partial adherence for a voluntary measure, then implementing an additional “top-down” NPI that reduces the transmission rate should be accompanied by a decrease in cost for the nonregulated measure to reduce the endemic level of infections. Such a decrease in cost could arise from easier access to the NPI (e.g., for masks, a lower cost or higher availability), or from incentives to nudge individuals (e.g., receiving other benefits if they adhere). Otherwise, due to behavioral feedback, the top-down measure may have no (or very little) effect on the number of infections in the long term.

**Fig. 2.**
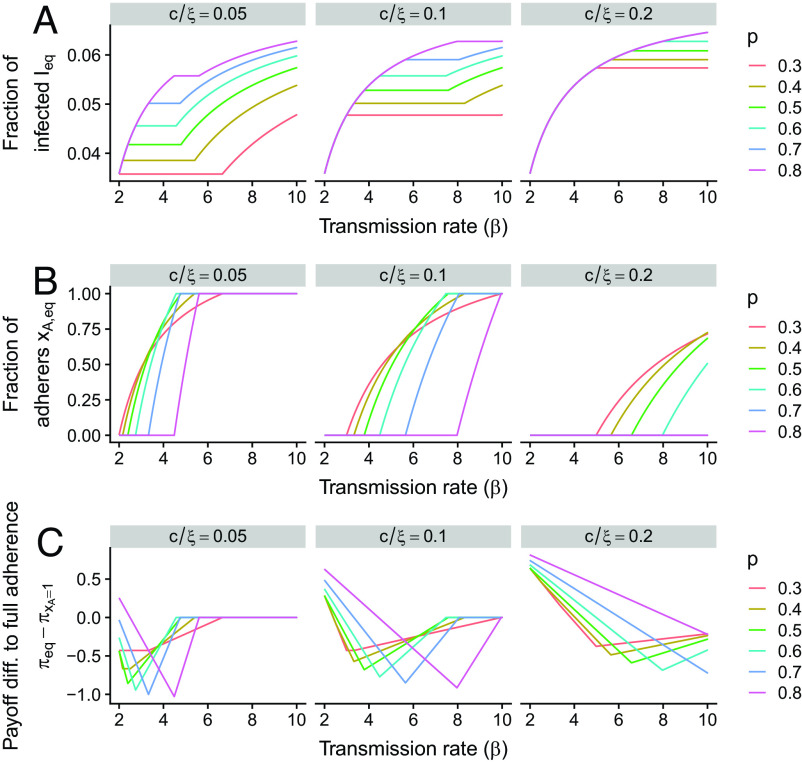
Impacts of the transmission rate (β per week) on the stable behavioral–epidemiological equilibrium for different cost-to-risk ratios $\frac{c}{\xi }$ and effectiveness of NPI p. (*A*) Equilibrium level of infections $I_{\mathrm{eq}}$. (*B*) Fraction of adherers $x_{A,eq}$. (*C*) payoff difference with complete adherence $\pi_{\mathrm{eq}}-\pi_{x_{A}=1}$. Other parameters are as follows: c= 1 per week, γ=1 per week, μ=0.02 per year, $\frac{1}{\delta }=0.25$ y. Note that values where the bifurcations occur, i.e., where one equilibrium gains stability while another loses it, are given in *SI Appendix*.

In Fig. 2, we illustrate the characteristics of the stable equilibrium as a function of the transmission rate. We examine a wide range of cost-to-risk ratios and effectivenesses of an NPI, and we assume immunity is short-lasting (0.25 y on average). For a high cost-to-risk ratio, some NPI adherence occurs in our chosen range of transmission rates only for very effective NPIs. When a transition to adherence occurs, the endemic level of infections becomes constant until complete adherence is reached. For less effective NPIs, there is zero adherence and the equilibrium endemic level of infections is correspondingly increasing as the transmission rate increases (Fig. 2 *A* and *B*, *Left Column*). As the cost-to-risk ratio decreases, the transitions to some and complete adherence occur for increasingly lower transmission rates (compare columns of Fig. 2 *A* and *B*). Furthermore, when there is partial adherence to the NPI and when the transmission rate is low, the fraction of adherers can be highest for a more effective NPI (lower p). However, as the transmission rate increases, less effective NPIs can lead to stronger adherence (Fig. 2*B*).

**Fig. 3.**
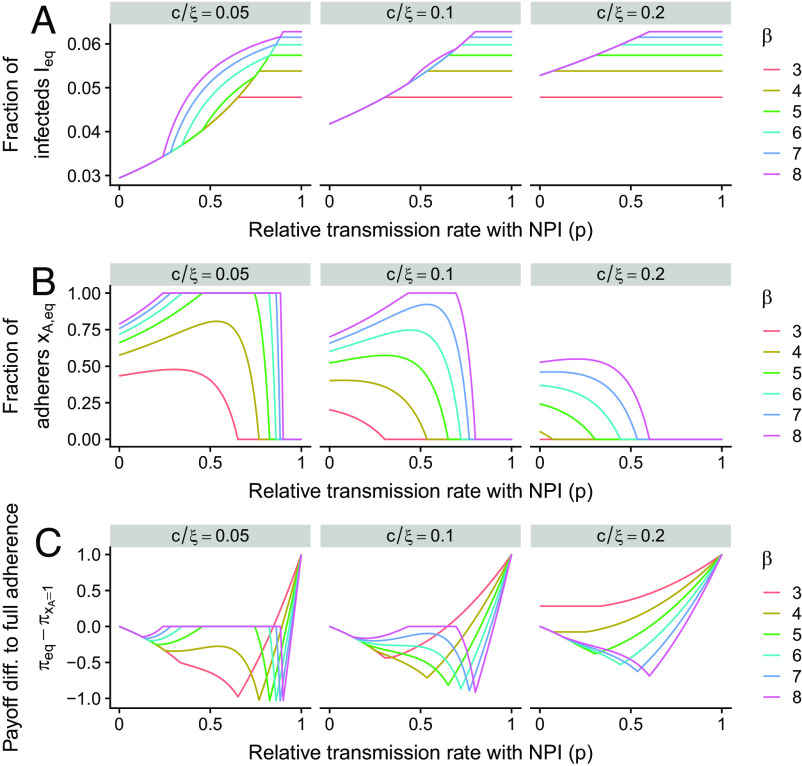
Effects of the NPI strength on the stable behavioral–epidemiological equilibrium. (*A*), (*B*), and (*C*) examine the levels of infection, adherence, and change in payoff relative to a completely adhering population, respectively, as a function of p, for different cost-to-risk ratios $\frac{c}{\xi }$ and for different transmission rates β (per week). Other parameters are as in Fig. 2.

In Fig. 3, we further investigate the dependence of the stable equilibrium on p. As the NPI decreases in effectiveness (increasing p), the fraction of infectious individuals at equilibrium can have substantial changes as a function of p (Fig. 3*A*). As either the transmission rate increases or the cost-to-risk ratio decreases, an intermediate effectiveness of NPI increases the fraction of adherers (Fig. 3*B*), and we explore this further in the *SI Appendix*. Finally, even if cost-to-risk ratios are low and transmission rates are high, as p approaches 1, i.e., the NPI is negligibly effective, there is a sharp transition to zero adherers (Fig. 3*B*, *Leftmost panel*).

### Comparisons with Complete Adherence to NPI.

In Figs. 2*C* and 3*C*, we examine the difference in payoff between the stable equilibrium and that of a population with complete adherence. Thus, a negative value means that the individual-level outcome penalizes the population and that complete adherence would be more favourable for the population. On the other hand, a positive value indicates that complete adherence would be less favourable for the population than the aggregate due to individual-level decision-making. For a higher cost-to-risk ratio, we find that this difference is positive when the transmission rate is low. As the transmission rate increases, the difference in payoff decreases and becomes negative; eventually, it begins to increase again and finally becomes zero (i.e., when complete adherence is the stable outcome). As either the cost-to-risk ratio decreases or the effectiveness of the NPI increases, these transitions occur for smaller transmission rates (Fig. 2*C*). These results are further emphasized in Fig. 3*C*. As the cost-to-risk ratio decreases, additional nonmonotonicities in the payoff difference emerge, where some intermediate values of p minimize the difference in payoff whereas others locally maximize it (at zero difference).

### Impact of Immunity.

So far, motivated by existing evidence for SARS-CoV-2, we have assumed that immunity is short-lasting. However, in the longer term, individual immunity to SARS-CoV-2 may accumulate with repeated exposures (e.g., scenarios explored in ref. 25). Alternatively, individual adherence to an NPI may not be driven by SARS-CoV-2, but in response to another pathogen with different characteristics. To explore these possibilities, we characterize the impacts of longer average durations of immunity on behavioral–epidemiological dynamics in our model.

**Fig. 4.**
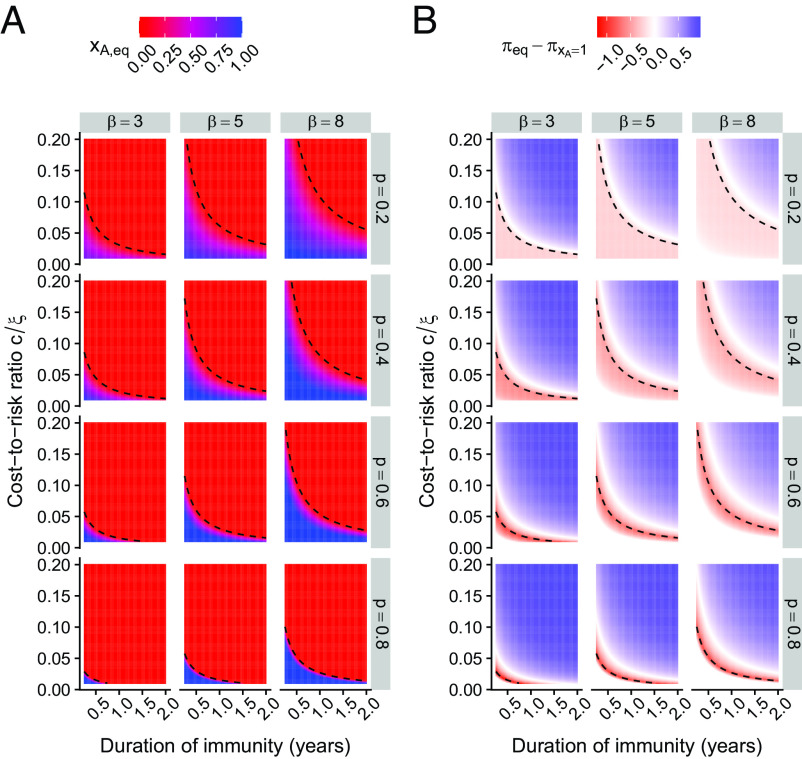
Effect of the duration of immunity on behavioral outcomes in our model. The (*A*) and (*B*) panels depict the fraction of adherers are equilibrium $x_{A,eq}$ and the relative payoff to a completely adhering population $\pi_{\mathrm{eq}}-\pi_{x_{A}=1}$, respectively, as a function of the duration of immunity ($\frac{1}{\delta })$ and the cost-to-risk ratio $\frac{c}{\xi }$, for different transmission rates β (per week) and effectiveness of NPI p. Other parameters are c=1 per week, γ=1 per week, μ=0.02 per year. In each plot, the dashed line illustrates where $\frac{c}{\xi }=\left(R_{0}-1\right)\left(1-p\right)\frac{\left(\gamma +\mu )(\mu +\delta \right)}{\gamma +\mu +\delta }$, i.e., $x_{A,\mathrm{eq}}=0$.

In Fig. 4, we examine the effect of the duration of immunity on behavioral dynamics. First, more durable immunity means that adherence requires increasingly lower cost-to-risk ratios. Intuitively, this effect is magnified by smaller transmission rates and less effective NPIs (compare plots of Fig. 4*A* from *Top Right* to *Bottom Left*). As previously, we compare in Fig. 4*B* the realized population payoff with that of a completely adhering population. Importantly, a longer period of immunity means that this difference is positive for increasingly smaller cost-to-risk ratios. Additionally, the most negative payoff differences occur for smaller cost-to-risk ratios. Thus, the duration of immunity can lead to important behavioral feedback, which affect individual adherence to an NPI.

### Effects of Vaccination on NPI Adherence and Disease Dyn-amics.

While our model framework focuses on NPIs, these control strategies are often used in conjunction with pharmaceutical interventions such as vaccination. In fact, as we have seen with SARS-CoV-2, large vaccination campaigns can trigger transitions from mandated NPIs to individual adherence decisions. Therefore, to examine the impacts of vaccination on the dynamics of NPI adherence and infection levels, we incorporate vaccination in our model both at birth and randomly (see *Materials and Methods* for model equations).

**Fig. 5.**
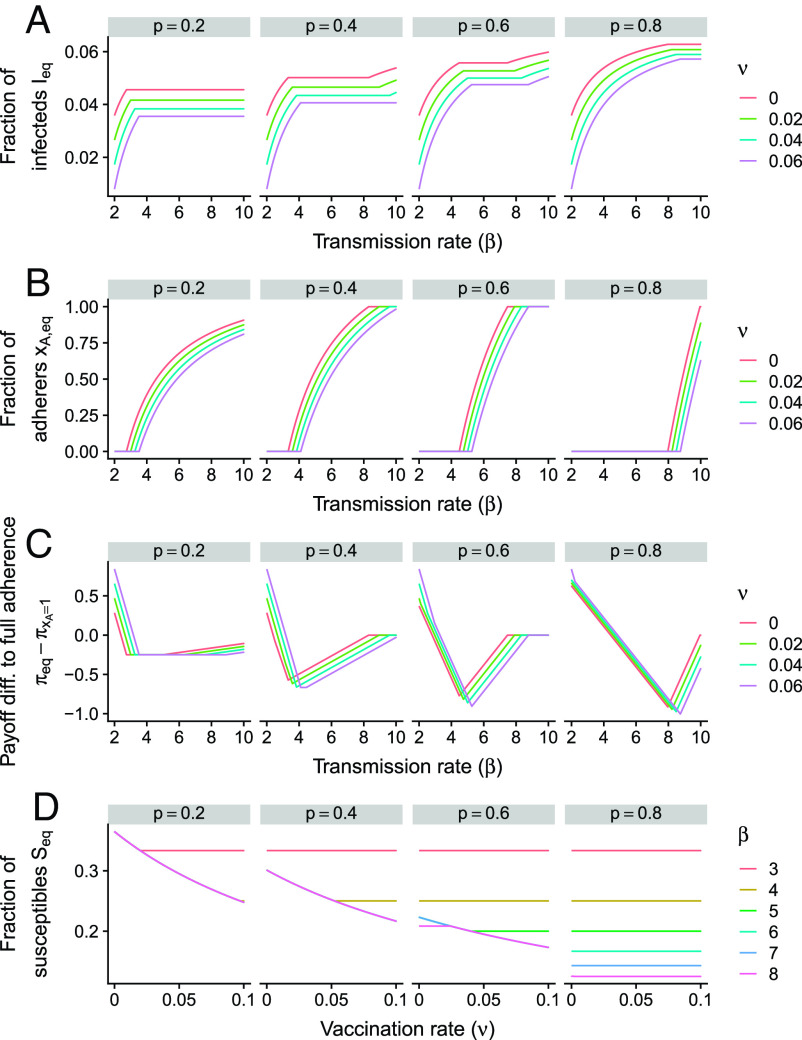
Impact of vaccination on behavioral–epidemiological dynamics. (*A*) Equilibrium characteristics as a function of the transmission rate β (per week), for different values of p and vaccination rates ν (per week). (*A*), (*B*), and (*C*) plot the equilibrium level of infections $I_{\mathrm{eq}}$, fraction of adherers $x_{A,eq}$, and difference in payoff compared to a fully-adhering population $\pi_{\mathrm{eq}}-\pi_{x_{A}=1}$, respectively. (*D*) Equilibrium level of susceptibles as a function of the vaccination rate ν (per week), for different values of p and transmission rates β (per week). Other parameters in *A*–*D* are $\frac{1}{\delta }=0.25$ y, ξ=10, c=1 per week, γ=1 per week, μ=0.02 per year, and q=0.

In our extended model with vaccination, there are analogous disease-free and endemic equilibria (*Materials and Methods*). While the endemic level of infections when there is partial adherence still does not depend on the transmission rate, vaccination impacts this quantity (the analytical expression is given in *Materials and Methods*). In Fig. 5*A*, we illustrate the stable equilibrium as a function of the transmission rate, for different vaccination rates and assuming that vaccination only occurs randomly (and not at birth, i.e., q=0). As the vaccination rate increases, the endemic level of infections decreases, even if a decrease in the transmission rate would have no impact. Thus, in this partial-adherence regime, an NPI that decreases the transmission rate may have a marginal impact on endemic infection levels but vaccination will successfully decrease infections. Echoing ref. 8, this result highlights the importance of vaccination for disease control.

Interestingly, as the vaccination rate increases, the equilibrium level of adherers decreases (Fig. 5*B*). Furthermore, for low transmission rates, vaccination increases the difference in payoff with a completely adhering population. On the other hand, for high transmission rates, vaccination decreases this difference (Fig. 5*C*). Thus, especially for high transmission rates, nudges to follow an NPI should yield even greater population-level benefits when vaccination is ongoing.

Finally, the equilibrium fraction of susceptible individuals in the partial-adherence regime decreases as vaccination increases (see *Materials and Methods* for this analytical expression). This relationship strongly contrasts with the SIRS model, where vaccination has no impact on the endemic level of susceptibles at equilibrium. For different values of p and transmission rates, Fig. 5*D* illustrates the change in the fraction of susceptible individuals at equilibrium as the vaccination rate ν increases. For NPIs that have a substantial effect on the transmission rate (low p), the level of susceptibles decreases with vaccination (unless the transmission rate is low). As the effectiveness of the NPI decreases (higher p), this dependence diminishes and is only apparent for increasingly higher transmission rates. Thus, our framework illustrates how NPI adherence decision-making can affect the vaccination susceptibility landscape.

## Caveats and Future Avenues

In our modelling framework, we have made a number of simplifying assumptions, which should be relaxed and investigated further in future work. First, we have ignored heterogeneities, such as those from transmission (32), age (33), or space, and these can substantially affect disease dynamics. Refining our modelling framework to examine their impacts on behavioral–epidemiological dynamics is therefore an important future direction.

We have also ignored heterogeneities in cost of adherence and perceived risk that the infection poses. Furthermore, both of these features may not be independent, e.g., some individuals who perceive a lower risk from the infection could also view adherence to an NPI as very costly. Relatedly, we have assumed that the benefit and cost of adherence to an NPI for an individual is independent of either their immune status or how recently they have been infected. However, it is possible that individuals who have been more recently infected (and deem themselves to be immune or at lower risk of infection) have a higher cost-to-risk ratio. Incorporating such behavioral heterogeneity is another important future direction.

Additionally, while we have included vaccination, we have assumed that vaccination occurs either at birth or randomly. However, vaccination decision-making may be an important factor that shapes both epidemic dynamics and individual costs of adherence to NPIs (34), and prior work has examined this interplay in detail (16, 17). Coupling both behavioral processes together and to an epidemiological model would be an especially valuable future avenue to pursue.

For simplicity, we have also modelled the impact of an NPI as the same whether either player is adhering. In reality, and as we have explored in prior work, NPIs may not be symmetric in this way, and the impact of the adherence of both players to an NPI may not be independent (20). Thus, future work should relax this assumption and examine the dynamical impacts of asymmetrical control strategies. Second, as we have discussed in prior work (20), individuals may have more than two choices for a given NPI (e.g., different face masks), and extending our model to include this would be particularly salient.

Relatedly, we have taken the simplifying assumption that the effectiveness of the NPI is fixed. However, if individuals that adhere change their adherence behavior as a reaction to the fraction of nonadherers (e.g., adhere less frequently to the NPI), the effectiveness of the NPI p could itself be time-dependent or depend on the fraction of nonadherers, and this is an important area for future work. Additionally, the situation where individuals are not continuously choosing whether to adhere or not should be explicitly investigated in future work.

In our work, we have also used a simple SIRS framework to model epidemiological dynamics and thus have assumed that individuals are either fully susceptible or fully immune. Recent immuno-epidemiological models (9, 23–25) have shown that the strength of immunity, i.e., relative susceptibility to reinfection (after waning of complete protection), is particularly important in shaping trajectories in the medium- and long-term. Thus, to examine the interplay between behavioral and immunity dynamics, future work should extend our framework to incorporate immuno-epidemiological models and also investigate the resulting dynamics if immunity and adherence are not independent. Relatedly, we have omitted gradual waning (e.g., ref. 35), and future work should also examine this. Furthermore, since immuno-epidemiology is closely tied to pathogen evolution (36–38), it would be interesting to also extend such a refined framework to examine the additional interplay between pathogen evolution and behavior.

Finally, we have assumed that ξ, the risk imposed by infection, is constant over time. However, this parameter could change due to various social and demographic factors. For example, this risk may decrease due to accumulating immunity (25), age-structure (33), or access to therapeutics (such as antivirals, e.g., ref. 39). On the other hand, ξ could increase due to an increased perception of risk from long-term consequences due to infection (e.g., Long COVID). Extending our modelling framework to include these factors would be important. Relatedly, while current decision-making of adherence to an NPI is shaped by SARS-CoV-2, optimistic long-term future characteristics of COVID-19 may affect this. In that case, individual NPI adherence could be governed by an ensemble of circulating (and emerging) pathogens, and extending our models to consider this setting would also be valuable.

## Conclusion

As we have seen throughout the SARS-CoV-2 pandemic, individual NPI adherence decision-making is complex and emerges from a number of important factors. These range from the (real or perceived) risk from infection to the cost of adherence, and are shaped by ongoing epidemiological dynamics. Thus, understanding the feedback between epidemiology and behavior, and characterizing the settings when individual and population incentives may not be aligned, are both crucial to design proper mitigation measures.

To investigate these dynamics, we have coupled epidemiological and behavioral scales for individual (“bottom-up”) NPI adherence in a simple model. Unexpectedly, we find that, in our model, when there is partial NPI adherence at equilibrium, a change in transmission rate has no impact on the endemic number of infections. This phenomenon emerges due to compensation from behavioral dynamics and illustrates the importance of strategic deployment for additional top-down NPIs. That is, to successfully decrease infection levels, such top-down strategies should be accompanied by incentives to encourage individual adherence to a bottom-up NPI.

Furthermore, our model highlights that vaccination can successfully decrease the number of infections at equilibrium, even if there is partial adherence to a bottom-up NPI. Thus, this finding illustrates another population-level benefit of vaccination (e.g., partially akin to the findings of ref. 8 for pathogens with high $R_{0}$). Finally, our simple model reveals parameter regions when individual incentives lead to tensions across scales. In these regions, nudging to follow an NPI would increase the population-level payoff and decrease such a tension.

## Materials and Methods

### A. Behavioral–Epidemiological Equilibrium Analyses.

#### A.1. Disease-free equilibria.

Our model, (3), has two disease-free equilibria, $P_{0}^{\left(0\right)}$ with zero (Theorem 1, *SI Appendix*), and $P_{0}^{\left(1\right)}$ with complete adherence (Theorem 1, *SI Appendix*). In our model, the basic reproduction number (in the absence of any NPI adherence) is $R_{0}=\frac{\beta }{\gamma +\mu }$. Examining the Jacobian matrix at each equilibrium shows that $P_{0}^{\left(0\right)}$ is locally asymptotically stable when $R_{0}<1$ and is unstable when $R_{0}>1$ (Theorem 1, *SI Appendix*). On the other hand, $P_{0}^{\left(1\right)}$ is always unstable (Theorem 1, *SI Appendix*).

#### A.2. Endemic equilibria.

The endemic equilibria in our model are as follows (Theorem 1, *SI Appendix*):

- $P_{*}^{\left(0\right)}$ with $x_{A,*}^{\left(0\right)}=0$ and $I_{*}^{\left(0\right)}=\frac{\mu +\delta }{\mu +\delta +\gamma }\left(1-\frac{1}{R_{0}}\right)$, $S_{*}^{\left(0\right)}=\frac{1}{R_{0}}$;
- $P_{*}^{\left(1\right)}$ with $x_{A,*}^{\left(1\right)}=1$ and $I_{*}^{\left(1\right)}=\frac{\mu +\delta }{\mu +\delta +\gamma }\left(1-\frac{1}{pR_{0}}\right)$, $S_{*}^{\left(1\right)}=\frac{1}{pR_{0}}$;
- $P_{*}^{\left(m\right)}$, where
  [4] $$\begin{array}{cc} I_{*}^{\left(m\right)} & =\frac{\mu +\delta }{\left(1-p\right)\left(\gamma +\mu \right)\left(\mu +\delta \right)\frac{\xi }{c}+\gamma +\mu +\delta }, \end{array}$$
  [5] $$\begin{array}{cc} S_{*}^{\left(m\right)} & =\frac{\left(\gamma +\mu \right)\left(\mu +\delta \right)\frac{\xi }{c}\left(1-p\right)}{\left(1-p\right)\frac{\xi }{c}\left(\gamma +\mu \right)\left(\mu +\delta \right)+\gamma +\mu +\delta }, \end{array}$$
  [6] $$\begin{array}{cc} x_{A}^{*} & =\frac{1}{1-p}\left[1-\frac{\gamma +\mu }{\beta }\left(1+\frac{1}{\left(1-p\right)\frac{\xi }{c}}\frac{\gamma +\mu +\delta }{\left(\gamma +\mu )(\mu +\delta \right)}\right)\right]. \end{array}$$

Intuitively, if the NPI is capable of decreasing the basic reproduction number below 1, i.e., $p<\frac{1}{R_{0}}$, then $P_{*}^{\left(1\right)}$ does not exist. Note also that $S_{*}^{\left(m\right)}$, $I_{*}^{\left(m\right)}$ and $x_{A}^{*}$ all only depend on the cost-to-risk ratio $\frac{c}{\xi }$, i.e., the cost of adhering to the measure relative to the risk posed by infection. Furthermore, the partial-adherence equilibrium $P_{*}^{\left(m\right)}$ only exists when $0<x_{A}^{*}<1$, which depends on the relative magnitude of $\frac{c}{\xi }$.

In Theorem 2 (*SI Appendix*), we prove that whenever the partial-adherence equilibrium $P_{*}^{\left(m\right)}$ exists, it is locally stable, and $P_{*}^{\left(0\right)}$ and $P_{*}^{\left(1\right)}$ (if the latter exists) are unstable. Additionally, provided $R_{0}>1$ and $p>\frac{1}{R_{0}}$, we show that whether $P_{*}^{\left(0\right)}$ or $P_{*}^{\left(1\right)}$ is stable depends on the relative magnitude of the ratio $\frac{c}{\xi }$ in relation to epidemiological parameters.

### B. Model with Vaccination.

#### B.1. Model equations.

As before, we denote $x_{A}$ as the fraction of individuals that adhere to the NPI. We further assume that vaccination can occur both at birth (i.e., a fraction q of new births are vaccinated) and randomly (at rate ν). In the latter case, we further assume that only susceptible individuals gain immune status (as in refs. 9, 24, and (23), giving

[7] $$\begin{array}{cc} \frac{\mathrm{dS}}{\mathrm{dt}} & =\mu \left(1-q\right)-[px_{A}+\left(1-x_{A}\right)]\beta SI-\mu S+\delta R-\nu S, \end{array}$$

[8] $$\begin{array}{cc} \frac{\mathrm{dI}}{\mathrm{dt}} & =\left[px_{A}+\left(1-x_{A}\right)\right]\beta IS-\left(\gamma +\mu \right)I, \end{array}$$

[9] $$\begin{array}{cc} \frac{\mathrm{dR}}{\mathrm{dt}} & =q\mu +\gamma I+\nu S-(\mu +\delta )R, \end{array}$$

[10] $$\begin{array}{cc} \frac{dx_{A}}{\mathrm{dt}} & =x_{A}\left(1-x_{A}\right)\left(\pi_{A}-\pi_{N}\right). \end{array}$$

#### B.2. Equilibrium analyses.

As before, since S+I+R=1, we examine the reduced system with R=1−S−I. In this model, the disease-free equilibria have $S=\frac{\mu (1-q)+\delta }{\mu +\nu +\delta }$, I=0, and either $x_{A}=0$ or $x_{A}=1$ (Theorem 3, *SI Appendix*). Thus, the basic reproduction number in this system is $R_{0}^{\left(v\right)}=\frac{\beta }{\gamma +\mu }\frac{\mu (1-q)+\delta }{\mu +\nu +\delta }$.

The endemic equilibria with zero ($x_{A}=0$) or complete adherence ($x_{A}=1$) have $S=\frac{\gamma +\mu }{\beta }$, $I=\frac{\mu (1-q)+\delta }{\gamma +\mu +\delta }\left(1-\frac{1}{R_{0}^{\left(v\right)}}\right)$ or $S=\frac{\gamma +\mu }{p\beta }$, $I=\frac{\mu (1-q)+\delta }{\gamma +\mu +\delta }\left(1-\frac{1}{pR_{0}^{\left(v\right)}}\right)$, respectively (Theorem 3, *SI Appendix*). (Again note that the complete adherence endemic equilibrium only exists if $p>\frac{1}{R_{0}^{\left(v\right)}}$.) As in the model with no vaccination, there is also an endemic equilibrium with partial adherence (Theorem 3, *SI Appendix*), given by

[11] $$\begin{array}{cc} I_{*}^{\left(m\right)} & =\frac{\mu (1-q)+\delta }{\left(1-p\right)\frac{\xi }{c}\left(\gamma +\mu \right)\left(\mu +\nu +\delta \right)+\gamma +\mu +\delta }, \end{array}$$

[12] $$\begin{array}{cc} S_{*}^{\left(m\right)} & =\frac{\left(\gamma +\mu \right)\left(\mu \left(1-q\right)+\delta \right)\frac{\xi }{c}\left(1-p\right)}{\left(1-p\right)\frac{\xi }{c}\left(\gamma +\mu \right)\left(\mu +\nu +\delta \right)+\gamma +\mu +\delta }, \end{array}$$

[13] $$\begin{array}{cc} x_{A}^{*} & =\frac{1}{1-p}\left[1-\frac{\mu +\nu +\delta }{\mu (1-q)+\delta }\frac{\gamma +\mu }{\beta }\left(1+\frac{1}{1-p}\frac{c}{\xi }\frac{\gamma +\mu +\delta }{\left(\gamma +\mu )(\mu +\nu +\delta \right)}\right)\right]. \end{array}$$

Theorem 4 (*SI Appendix*) addresses the (analogous) stability of equilibria in the expanded model with vaccination.

## Supplementary Material

Appendix 01 (PDF)

## Data Availability

Codes are available as *SI Appendix* files.
