# Supplementary material for: Dynamics in a behavioral–epidemiological model for individual adherence to a nonpharmaceutical intervention
Source: Proc Natl Acad Sci U S A. 2023 Oct 27;120(44):e2311584120. doi: 10.1073/pnas.2311584120 (PMC10622941; doi:10.1073/pnas.2311584120)
Supplement: Supplementary file 1 — Appendix 01 (PDF) [file pnas.2311584120.sapp.pdf]

# Supporting Information for

## Dynamics in a behavioral-epidemiological model for individual adherence to a nonpharmaceutical intervention

**Chadi M. Saad-Roy and Arne Traulsen**

**Chadi M. Saad-Roy.**
**E-mail: csaadroy@berkeley.edu**

**This PDF file includes:**

Supporting text

## Supporting Information Text

## Theorems and proofs

### Equilibria of the system.

**Theorem 1.** *The possible equilibria in our behavioral-epidemiological model are as follows.*

1. *There is a disease-free equilibrium with no adherence* $P_0^{(0)}$*, with* $S_0 = 1$*,* $I_0 = 0$ *and* $x_{A,0} = 0$*.*
2. *There is a disease-free equilibrium with complete adherence* $P_0^{(1)}$*, with* $S_0^{(1)} = 1$*,* $I_0^{(1)} = 0$*, and* $x_{A,0}^{(1)} = 1$*.*
3. *Suppose* $\mathcal{R}_0 > 1$*.*
   (a) *There is an endemic equilibrium with no adherence* $P_*^{(0)}$ *that exists, with* $x_{A,*}^{(0)} = 0$*,* $I_*^{(0)} = \frac{\mu+\delta}{\gamma+\mu+\delta}\left(1 - \frac{\gamma+\mu}{\beta}\right)$*, and* $S_*^{(0)} = \frac{\gamma+\mu}{\beta}$*.*
   (b) *If* $p > \frac{1}{\mathcal{R}_0}$*, then there is an endemic equilibrium with complete adherence* $P_*^{(1)}$ *that exists, with* $x_{A,*}^{(1)} = 1$*,* $I_*^{(1)} = \frac{\mu+\delta}{\gamma+\mu+\delta}\left(1 - \frac{\gamma+\mu}{p\beta}\right)$*, and* $S_*^{(0)} = \frac{\gamma+\mu}{p\beta}$*.*
   (c) *If either*
      i. $p > \frac{1}{\mathcal{R}_0}$ *and* $p(1-p)\beta\frac{\mu+\delta}{\gamma+\mu+\delta}\left(1 - \frac{\gamma+\mu}{p\beta}\right) < \frac{c}{\xi} < (1-p)\beta\frac{\mu+\delta}{\gamma+\mu+\delta}\left(1 - \frac{\gamma+\mu}{\beta}\right)$*, or*
      ii. $p < \frac{1}{\mathcal{R}_0}$ *and* $\frac{c}{\xi} < (1-p)\beta\frac{\mu+\delta}{\gamma+\mu+\delta}\left(1 - \frac{\gamma+\mu}{\beta}\right)$*,*

   *then there is an endemic equilibrium with partial adherence* $P_*^{(m)}$ *that exists, with*

$$x_A^* = \frac{1}{1-p}\left[1 - \frac{\gamma+\mu}{\beta}\left(1 + \frac{1}{(1-p)\frac{\xi}{c}}\frac{\gamma+\mu+\delta}{(\gamma+\mu)(\mu+\delta)}\right)\right], \quad [1]$$

$$I_*^{(m)} = \frac{\mu+\delta}{(1-p)(\gamma+\mu)(\mu+\delta)\frac{\xi}{c} + \gamma+\mu+\delta}, \quad [2]$$

$$S_*^{(m)} = \frac{(\gamma+\mu)(\mu+\delta)\frac{\xi}{c}(1-p)}{(1-p)\frac{\xi}{c}(\gamma+\mu)(\mu+\delta) + \gamma+\mu+\delta}. \quad [3]$$

*Proof.* We begin by proving $\{1\}$ and $\{3\ (a)\}$. Setting model equations (see Main Text) equal to zero, we see that $x_A = x_{A,*}^{(0)} = 0$ satisfies $\frac{dx_A}{dt} = 0$, in turn giving a regular SIRS model with transmission rate $\beta$. Thus, solving the remaining equations gives that either $I = I_0 = 0$ and $S = S_0 = 1$, or $I = I_*^{(0)} = \frac{\mu+\delta}{\gamma+\mu+\delta}\left(1 - \frac{\gamma+\mu}{\beta}\right)$ and $S = S_*^{(0)} = \frac{1}{\mathcal{R}_0}$. Note that $I_*^{(0)}$ and $S_*^{(0)}$ exist in the feasible region only when $\mathcal{R}_0 > 1$.

Similarly (to prove $\{2\}$ and $\{3\ (b)\}$), if $x_A = x_{A,*}^{(1)} = 1$, $\frac{dx_A}{dt} = 0$, which leaves a regular SIRS model with transmission rate $p\beta$ and corresponding basic reproduction number $p\mathcal{R}_0$. As before, the $S$ and $I$ equations give that either $I = I_0^{(1)} = 0$ and $S = S_0^{(1)} = 1$, or $I = I_*^{(1)} = \frac{\mu+\delta}{\gamma+\mu+\delta}\left(1 - \frac{\gamma+\mu}{p\beta}\right)$ and $S = S_*^{(1)} = \frac{1}{p\mathcal{R}_0}$. Note that this latter equilibrium exists in the feasible region only when $p\mathcal{R}_0 > 1$, *i.e.* $\mathcal{R}_0 > 1$ and $p > \frac{1}{\mathcal{R}_0}$.

Furthermore (to prove $\{3\ (c)\}$), when $\pi_A - \pi_N = 0$, then $\frac{dx_A}{dt} = 0$. Solving $\pi_A - \pi_N = 0$ with $\mu - [px_A + (1-x_A)]\beta SI - \mu S + \delta(1 - S - I) = 0$ and $[px_A + (1-x_A)]\beta SI - (\gamma+\mu)I = 0$ gives Eqs. [1]–[3]. Using the fact that $0 < x_A^* < 1$ gives the inequalities in $(c)$ (and noting that $c > 0$, for $\{(c)ii\}$). □

### Stability.

**Theorem 2.** *The local stability of the possible equilibria are as follows:*

1. $P_0^{(0)}$ *is locally asymptotically stable when* $\mathcal{R}_0 < 1$ *and unstable when* $\mathcal{R}_0 > 1$*.*
2. $P_0^{(1)}$ *is always unstable.*
3. *Suppose* $\mathcal{R}_0 > 1$*.*
   (a) *If* $\frac{c}{\xi} > (1-p)\beta\frac{\mu+\delta}{\gamma+\mu+\delta}\left(1 - \frac{\gamma+\mu}{\beta}\right)$*, then* $P_*^{(0)}$ *is locally asymptotically stable, whereas it is unstable if* $\frac{c}{\xi} < (1-p)\beta\frac{\mu+\delta}{\gamma+\mu+\delta}\left(1 - \frac{\gamma+\mu}{\beta}\right)$*.*
   (b) *Suppose also that* $p > \frac{1}{\mathcal{R}_0}$*:*

*i. If $\frac{c}{\xi} < p(1-p)\beta\frac{\mu+\delta}{\gamma+\mu+\delta}\left(1-\frac{\gamma+\mu}{p\beta}\right)$, then $P_*^{(1)}$ is locally asymptotically stable, whereas it is unstable if $\frac{c}{\xi} > p(1-p)\beta\frac{\mu+\delta}{\gamma+\mu+\delta}\left(1-\frac{\gamma+\mu}{p\beta}\right)$.*

*ii. If $p(1-p)\beta\frac{\mu+\delta}{\gamma+\mu+\delta}\left(1-\frac{\gamma+\mu}{p\beta}\right) < \frac{c}{\xi} < (1-p)\beta\frac{\mu+\delta}{\gamma+\mu+\delta}\left(1-\frac{\gamma+\mu}{\beta}\right)$, then $P_*^{(m)}$ is locally asymptotically stable.*

*(c) On the other hand, suppose that $p < \frac{1}{\mathcal{R}_0}$:*

*i. If $\frac{c}{\xi} < (1-p)\beta\frac{\mu+\delta}{\gamma+\mu+\delta}\left(1-\frac{\gamma+\mu}{\beta}\right)$, then $P_*^{(m)}$ is locally asymptotically stable (and $P_*^{(0)}$ is unstable)*

*Proof.* First, note that the Jacobian matrix about any equilibrium $\widehat{P}$ is

$$J(\widehat{P}) = \begin{pmatrix} -\beta\widehat{I}(1-(1-p)\widehat{x}_A) - (\mu+\delta) & -\beta\widehat{S}(1-(1-p)\widehat{x}_A) - \delta & (1-p)\beta\widehat{I}\widehat{S} \\ \beta\widehat{I}(1-(1-p)\widehat{x}_A) & (1-(1-p)\widehat{x}_A)\beta\widehat{S} - (\gamma+\mu) & -(1-p)\beta\widehat{S}\widehat{I} \\ 0 & \widehat{x}_A(1-\widehat{x}_A)(1-p)\beta\xi(1-(1-p)\widehat{x}_A) & (1-2\widehat{x}_A)(\pi_A-\pi_N) - \widehat{x}_A(1-\widehat{x}_A)(1-p)^2\beta\widehat{I}\xi \end{pmatrix}. \quad [4]$$

We begin by proving {1}. The Jacobian matrix for $P_0^{(0)}$ is

$$J(P_0^{(0)}) = \begin{pmatrix} -(\mu+\delta) & -\beta-\delta & 0 \\ 0 & \beta-(\gamma+\mu) & 0 \\ 0 & 0 & -c \end{pmatrix}. \quad [5]$$

Since the eigenvalues are $-(\mu+\delta) < 0$, $-c < 0$, and $\beta - (\gamma+\mu)$, $P_0^{(0)}$ is stable when $\mathcal{R}_0 = \frac{\beta}{\gamma+\mu} < 1$ and unstable when $\mathcal{R}_0 > 1$.

To prove {2}, the Jacobian matrix for $P_0^{(1)}$ is

$$J(P_0^{(1)}) = \begin{pmatrix} -(\mu+\delta) & -p\beta-\delta & 0 \\ 0 & \beta-(\gamma+\mu) & 0 \\ 0 & 0 & c \end{pmatrix}. \quad [6]$$

Since one of the eigenvalues of $J(P_0^{(1)})$ is $c > 0$, then $P_0^{(1)}$ is always unstable.

To prove {3}, we begin by examining the $\{(a)\}$ case. The Jacobian matrix at $P_*^{(0)}$ is

$$J(P_*^{(0)}) = \begin{pmatrix} -\beta I_*^{(0)} - (\mu+\delta) & -\beta S_*^{(0)} - \delta & (1-p)\beta I_*^{(0)} S_*^{(0)} \\ \beta I_*^{(0)} & 0 & -(1-p)\beta S_*^{(0)} I_*^{(0)} \\ 0 & 0 & (1-p)\beta I_*^{(0)}\xi - c \end{pmatrix}. \quad [7]$$

Since $J(P_*^{(0)})$ is block diagonal, two eigenvalues of $J(P_*^{(0)})$ are those of the matrix

$$A = \begin{pmatrix} -\beta I_*^{(0)} - (\mu+\delta) & -\beta S_*^{(0)} - \delta \\ \beta I_*^{(0)} & 0 \end{pmatrix}. \quad [8]$$

$A$ has a negative trace and a positive determinant, and so both of its eigenvalues have negative real part by the Routh-Hurwitz criterion. The third eigenvalue of $J(P_*^{(0)})$ is $(1-p)\beta I_*^{(0)}\xi - c$, which is negative when $c > (1-p)\beta I_*^{(0)}\xi$. Thus, if $c > (1-p)\beta\frac{\mu+\delta}{\gamma+\mu+\delta}\left(1-\frac{\gamma+\mu}{\beta}\right)\xi$, then $P_*^{(0)}$ is locally asymptotically stable, whereas if $c > (1-p)\beta\frac{\mu+\delta}{\gamma+\mu+\delta}\left(1-\frac{\gamma+\mu}{\beta}\right)\xi$, $P_*^{(0)}$ is unstable.

To prove the $\{(b)i\}$ case, consider the Jacobian matrix

$$J(P_*^{(1)}) = \begin{pmatrix} -p\beta I_*^{(1)} - (\mu+\delta) & -p\beta S_*^{(0)} - \delta & (1-p)\beta I_*^{(1)} S_*^{(1)} \\ p\beta I_*^{(1)} & 0 & -(1-p)\beta I_*^{(1)} S_*^{(1)} \\ 0 & 0 & -p(1-p)\beta I_*^{(1)}\xi + c \end{pmatrix}. \quad [9]$$

As previously, two eigenvalues of $J(P_*^{(1)})$ are eigenvalues of

$$B = \begin{pmatrix} -p\beta I_*^{(1)} - (\mu+\delta) & -p\beta S_*^{(0)} - \delta \\ p\beta I_*^{(1)} & 0 \end{pmatrix}. \quad [10]$$

Since $tr(B) < 0$ and $\det(B) > 0$, it follows that both eigenvalues of $B$ have negative real part. The last eigenvalue of $J(P_*^{(1)})$ is $-p(1-p)\beta I_*^{(1)}\xi + c$, which is negative when $c < p(1-p)\beta\frac{\mu+\delta}{\gamma+\mu+\delta}\left(1-\frac{\gamma+\mu}{p\beta}\right)\xi$. Thus, $P_*^{(1)}$ is locally asymptotically stable when $c < p(1-p)\beta\frac{\mu+\delta}{\gamma+\mu+\delta}\left(1-\frac{\gamma+\mu}{p\beta}\right)\xi$, whereas it is unstable when $c > p(1-p)\beta\frac{\mu+\delta}{\gamma+\mu+\delta}\left(1-\frac{\gamma+\mu}{p\beta}\right)\xi$.

We now prove the $\{(b)ii\}$ and $\{(c)i\}$ cases. For $P_*^{(m)}$, the Jacobian matrix is

$$J(P_*^{(m)}) = \begin{pmatrix} -\beta I_*^{(m)}(1-(1-p)x_A^*) - (\mu+\delta) & -\beta S_*^{(m)}(1-(1-p)x_A^*) - \delta & (1-p)\beta I_*^{(m)} S_*^{(m)} \\ \beta I_*^{(m)}(1-(1-p)x_A^*) & 0 & -(1-p)\beta S_*^{(m)} I_*^{(m)} \\ 0 & x_A^*(1-x_A^*)(1-p)\beta\xi(1-(1-p)x_A^*) & -x_A^*(1-x_A^*)(1-p)^2\beta I_*^{(m)}\xi \end{pmatrix} \quad [11]$$

Thus,

$$tr(J(P_*^{(m)})) = -\beta I_*^{(m)}(1-(1-p)x_A^*) - (\mu+\delta) - x_A^*(1-x_A^*)(1-p)^2\beta I_*^{(m)}\xi. \quad [12]$$

Computing the determinant gives that

$$\begin{aligned}\det(J(P_*^{(m)})) =& -\beta I_*^{(m)}(1-(1-p)x_A^*)(\beta S_*^{(m)}(1-(1-p)x_A^*) + \delta)x_A^*(1-x_A^*)(1-p)^2\beta I_*^{(m)}\xi \\ & - (\mu+\delta)(1-p)\beta S_*^{(m)} I_*^{(m)} x_A^*(1-x_A^*)(1-p)\beta\xi(1-(1-p)x_A^*). \end{aligned} \quad [13]$$

Computing the sum of the $2\times 2$ principal minors of $J(P_*^{(m)})$ gives that

$$\begin{aligned} a_2 =& \beta I_*^{(m)}(1-(1-p)x_A^*)[\beta S_*^{(m)}(1-(1-p)x_A^*) + \delta] \\ & + [\beta I_*^{(m)}(1-(1-p)x_A^*) + \mu + \delta]x_A^*(1-x_A^*)(1-p)^2\beta I_*^{(m)}\xi \\ & + (1-p)\beta S_*^{(m)} I_*^{(m)} x_A^*(1-x_A^*)(1-p)\beta\xi(1-(1-p)x_A^*). \end{aligned} \quad [14]$$

Note that whenever $P_*^{(m)}$ exists, $x_A^*$ is such that $0 < x_A^* < 1$. Thus, when $P_*^{(m)}$ exists, $tr(J(P_*^{(m)})) < 0$, $\det(J(P_*^{(m)})) < 0$, and it can be seen that $tr(J(P_*^{(m)}))a_2 - \det(J(P_*^{(m)})) < 0$. Therefore, by the Routh-Hurwitz criterion, $P_*^{(m)}$ is locally asymptotically stable whenever it exists, *i.e.*, when

$$p(1-p)\beta\frac{\mu+\delta}{\gamma+\mu+\delta}\left(1-\frac{\gamma+\mu}{p\beta}\right) < \frac{c}{\xi} < (1-p)\beta\frac{\mu+\delta}{\gamma+\mu+\delta}\left(1-\frac{\gamma+\mu}{\beta}\right) \quad \text{if} \quad p > \frac{1}{\mathcal{R}_0} \quad [15]$$

$$\frac{c}{\xi} < (1-p)\beta\frac{\mu+\delta}{\gamma+\mu+\delta}\left(1-\frac{\gamma+\mu}{\beta}\right) \quad \text{if} \quad p < \frac{1}{\mathcal{R}_0}. \quad [16]$$

□

**Vaccination.**

**Theorem 3.** *With vaccination, the possible equilibria are analogous to those without vaccination, but with*

- *the disease-free equilibria* $P_0^{(0)}$ *and* $P_0^{(1)}$ *having instead* $S_0 = \frac{\mu(1-q)+\delta}{\mu+\nu+\delta}$*;*
- *the endemic equilibria* $P_*^{(0)}$ *and* $P_*^{(1)}$ *having instead, respectively,* $I_*^{(0)} = \frac{\mu(1-q)+\delta}{\gamma+\mu+\delta}\left(1-\frac{1}{\mathcal{R}_0^{(v)}}\right)$ *and* $I_*^{(1)} = \frac{\mu(1-q)+\delta}{\gamma+\mu+\delta}\left(1-\frac{1}{p\mathcal{R}_0^{(v)}}\right)$*;*
- *and the endemic equilibrium with partial adherence* $P_*^{(m)}$ *having instead*

$$I_*^{(m)} = \frac{\mu(1-q)+\delta}{(1-p)\frac{\xi}{c}(\gamma+\mu)(\mu+\nu+\delta) + \gamma+\mu+\delta}, \quad [17]$$

$$S_*^{(m)} = \frac{(\gamma+\mu)(\mu(1-q)+\delta)\frac{\xi}{c}(1-p)}{(1-p)\frac{\xi}{c}(\gamma+\mu)(\mu+\nu+\delta) + \gamma+\mu+\delta}, \quad [18]$$

$$x_A^* = \frac{1}{1-p}\left[1 - \frac{\mu+\nu+\delta}{\mu(1-q)+\delta}\frac{\gamma+\mu}{\beta}\left(1 + \frac{1}{1-p}\frac{c}{\xi}\frac{\gamma+\mu+\delta}{(\gamma+\mu)(\mu+\nu+\delta)}\right)\right]. \quad [19]$$

[20]

*Note also that the conditions change so that* $P_*^{(1)}$ *exists when* $p > \frac{1}{\mathcal{R}_0^{(v)}}$*, and* $P_*^{(m)}$ *exists when either* $p > \frac{1}{\mathcal{R}_0^{(v)}}$ *and* $p(1-$

$p)\beta\frac{\mu(1-q)+\delta}{\gamma+\mu+\delta}\left(1-\frac{1}{p\mathcal{R}_0^{(v)}}\right) < \frac{c}{\xi} < (1-p)\beta\frac{\mu(1-q)+\delta}{\gamma+\mu+\delta}\left(1-\frac{1}{\mathcal{R}_0^{(v)}}\right)$ *or* $p < \frac{1}{\mathcal{R}_0^{(v)}}$ *and* $\frac{c}{\xi} < (1-p)\beta\frac{\mu(1-q)+\delta}{\gamma+\mu+\delta}\left(1-\frac{1}{\mathcal{R}_0^{(v)}}\right)$.

*Proof.* This result follows from using the equations at equilibrium in the model with vaccination, and following the same approach as in Theorem 1 (*SI Appendix*). □

**Theorem 4.** *With vaccination, the stability of each equilibria is analogous to that in the model without vaccination, but the conditions change so that* $\mathcal{R}_0^{(v)}$ *is used instead of* $\mathcal{R}_0$ *and* $\frac{\mu(1-q)+\delta}{\gamma+\mu+\delta}$ *is used instead of* $\frac{\mu+\delta}{\gamma+\mu+\delta}$.

*Proof.* The Jacobian matrix for any equilibrium $\widehat{P} = (\widehat{S}, \widehat{I}, \widehat{x}_A)$ in the model with vaccination is

$$J(\widehat{P}) = \begin{pmatrix} -\beta\widehat{I}(1-(1-p)\widehat{x}_A) - (\mu+\nu+\delta) & -\beta\widehat{S}(1-(1-p)\widehat{x}_A) - \delta & (1-p)\beta\widehat{I}\widehat{S} \\ \beta\widehat{I}(1-(1-p)\widehat{x}_A) & (1-(1-p)x_A)\beta\widehat{S} - (\gamma+\mu) & -(1-p)\beta\widehat{S}\widehat{I} \\ 0 & \widehat{x}_A(1-\widehat{x}_A)(1-p)\beta\xi(1-(1-p)\widehat{x}_A) & (1-2\widehat{x}_A)(\pi_A-\pi_N) - \widehat{x}_A(1-\widehat{x}_A)(1-p)^2\beta\widehat{I}\xi \end{pmatrix} \quad [21]$$

□

At $P_0^{(1)}$, it then follows that

$$J(P_0^{(1)}) = \begin{pmatrix} -(\mu+\nu+\delta) & -p\beta S_0^{(1)} - \delta & 0 \\ 0 & p\beta {S_0}^{(1)} - (\gamma+\mu) & 0 \\ 0 & 0 & c \end{pmatrix}. \quad [22]$$

As without vaccination, since $c > 0$, $P_0^{(1)}$ is always unstable.

At $P_0^{(0)}$, it follows that

$$J(P_0^{(0)}) = \begin{pmatrix} -(\mu+\nu+\delta) & -\beta S_0^{(0)} - \delta & 0 \\ 0 & \beta {S_0}^{(0)} - (\gamma+\mu) & 0 \\ 0 & 0 & -c \end{pmatrix}, \quad [23]$$

so that the eigenvalues are $-c < 0$, $-(\mu+\nu+\delta) < 0$, and $\beta S_0^{(0)} - (\gamma+\mu)$. Note that $\beta S_0^{(0)} - (\gamma+\mu) < 0$ if $\mathcal{R}_0^{(v)} < 1$, and $\beta S_0^{(0)} - (\gamma+\mu) > 0$ if $\mathcal{R}_0^{(v)} > 1$. Thus, $P_0^{(0)}$ is locally asymptotically stable if $\mathcal{R}_0^{(v)} < 1$, and $P_0^{(0)}$ is unstable if $\mathcal{R}_0^{(v)} > 1$.

At $P_*^{(0)}$, the Jacobian matrix is

$$J(P_*^{(0)}) = \begin{pmatrix} -\beta I_*^{(0)} - (\mu+\nu+\delta) & -\beta S_*^{(0)} - \delta & (1-p)\beta I_*^{(0)} S_*^{(0)} \\ \beta I_*^{(0)} & 0 & -(1-p)\beta S_*^{(0)} I_*^{(0)} \\ 0 & 0 & -(1-p)\beta I_*^{(0)}\xi + c \end{pmatrix} \quad [24]$$

Thus, two eigenvalues are those of the $2 \times 2$ matrix

$$\begin{pmatrix} -\beta I_*^{(0)} - (\mu+\nu+\delta) & -\beta S_*^{(0)} - \delta \\ \beta I_*^{(0)} & 0 \end{pmatrix} \quad [25]$$

which has negative trace and positive determinant. Therefore, both of these eigenvalues have negative real part. The third eigenvalue of $J(P_*^{(0)})$ is $-(1-p)\beta I_*^{(0)}\xi + c$, which is negative when $c < (1-p)\beta\frac{\mu(1-q)+\delta}{\mu+\nu+\delta}\left(1-\frac{1}{\mathcal{R}_0^{(v)}}\right)\xi$. Thus, it follows that $P_*^{(0)}$ is locally asymptotically stable when $\frac{c}{\xi} < (1-p)\beta\frac{\mu(1-q)+\delta}{\mu+\nu+\delta}\left(1-\frac{1}{\mathcal{R}_0^{(v)}}\right)$.

At $P_*^{(1)}$, the Jacobian matrix is

$$J(P_*^{(1)}) = \begin{pmatrix} -p\beta I_*^{(1)} - (\mu+\nu+\delta) & -p\beta S_*^{(1)} - \delta & (1-p)\beta I_*^{(1)} S_*^{(1)} \\ p\beta I_*^{(1)} & 0 & -(1-p)\beta S_*^{(1)} I_*^{(1)} \\ 0 & 0 & (1-p)p\beta I_*^{(1)}\xi - c \end{pmatrix} \quad [26]$$

Note that the matrix

$$\begin{pmatrix} -p\beta I_*^{(1)} - (\mu+\nu+\delta) & -p\beta S_*^{(1)} - \delta \\ p\beta I_*^{(1)} & 0 \end{pmatrix} \qquad [27]$$

has negative trace and positive determinant, and that $(1-p)p\beta I_*^{(1)}\xi - c$ is negative if $c > (1-p)p\beta \frac{\mu(1-q)+\delta}{\mu+\gamma+\delta}\left(1 - \frac{1}{p\mathcal{R}_0^{(v)}}\right)\xi$. Thus, analogous to the case without vaccination, $P_*^{(1)}$ is locally asymptotically stable if $\frac{c}{\xi} > (1-p)p\beta \frac{\mu(1-q)+\delta}{\mu+\gamma+\delta}\left(1 - \frac{1}{p\mathcal{R}_0^{(v)}}\right)$.

For $P_*^{(m)}$, the Jacobian matrix is

$$J(P_*^{(m)}) = \begin{pmatrix} -\beta I_*^{(m)}(1-(1-p)x_A^*) - (\mu+\nu+\delta) & -\beta S_*^{(m)}(1-(1-p)x_A^*) - \delta & (1-p)\beta I_*^{(m)} S_*^{(m)} \\ \beta I_*^{(m)}(1-(1-p)x_A^*) & 0 & -(1-p)\beta S_*^{(m)} I_*^{(m)} \\ 0 & x_A^*(1-x_A^*)(1-p)\beta\xi(1-(1-p)x_A^*) & -x_A^*(1-x_A^*)(1-p)^2\beta I_*^{(m)}\xi \end{pmatrix}. \qquad [28]$$

Similar calculations to the analysis without vaccination shows that $tr(J(P_*^{(m)})) < 0$, $\det(J(P_*^{(m)})) < 0$, and $tr(J(P_*^{(m)}))a_2 - \det(J(P_*^{(m)})) < 0$ when $P_*^{(m)}$ exists, *i.e.*, $0 < x_A^* < 1$. Thus, as in the model without vaccination, $P_*^{(m)}$ is locally asymptotically stable whenever it exists.

## Maximal adherence to the NPI

In the model with no vaccination, if there is no complete adherence for the range of $p$, *i.e.* the maximum adherence happens with the partial equilibrium, then we can solve $\frac{\partial x_A^*}{\partial p} = 0$, giving

$$\widehat{p} = 1 - \frac{2\frac{\gamma+\mu}{\beta}\frac{c}{\xi}\frac{\gamma+\mu+\delta}{(\gamma+\mu)(\mu+\delta)}}{1 - \frac{\gamma+\mu}{\beta}}. \qquad [29]$$
